# Molecular mechanisms of dendrite morphogenesis

**DOI:** 10.3389/fncel.2012.00061

**Published:** 2012-12-28

**Authors:** Jyothi Arikkath

**Affiliations:** Developmental Neuroscience, Munroe-Meyer Institute, University of Nebraska Medical CenterOmaha, NE, USA

**Keywords:** dendrite, molecular mechanims, arborization, aberrant dendritic morphology, plasticity and learning

## Abstract

Dendrites are key integrators of synaptic information in neurons and play vital roles in neuronal plasticity. Hence, it is necessary that dendrite arborization is precisely controlled and coordinated with synaptic activity to ensure appropriate functional neural network integrity. In the past several years, it has become increasingly clear that several cell intrinsic and extrinsic mechanisms contribute to dendritic arborization. In this review, we will discuss some of the molecular mechanisms that regulate dendrite morphogenesis, particularly in cortical and hippocampal pyramidal neurons and some of the implications of aberrant dendritic morphology for human disease. Finally, we will discuss the current challenges and future directions in the field.

## Introduction

Neurons are highly specialized and polarized cells that have morphologically distinct regions optimized for their functional roles. Most neurons have several dendrites that represent the information input centers of the neuron and single axons that represent the information output centers. The majority of our understandings of the cellular and molecular mechanisms that control neuronal morphology have come from studies on the pyramidal neurons, which represent the vast majority of excitatory neurons in the hippocampus and cortex. In this review, we will focus on some of the more recent studies that have provided critical insights into the molecular and cellular mechanisms that control neuronal morphology, with particular reference to dendrites of pyramidal neurons in the hippocampus and cortex. In addition, we will review the consequences of aberrations in dendritic arborization in disease. Finally, we will discuss the current challenges in the field.

## Dendritic structure and function

**Figure 1:**
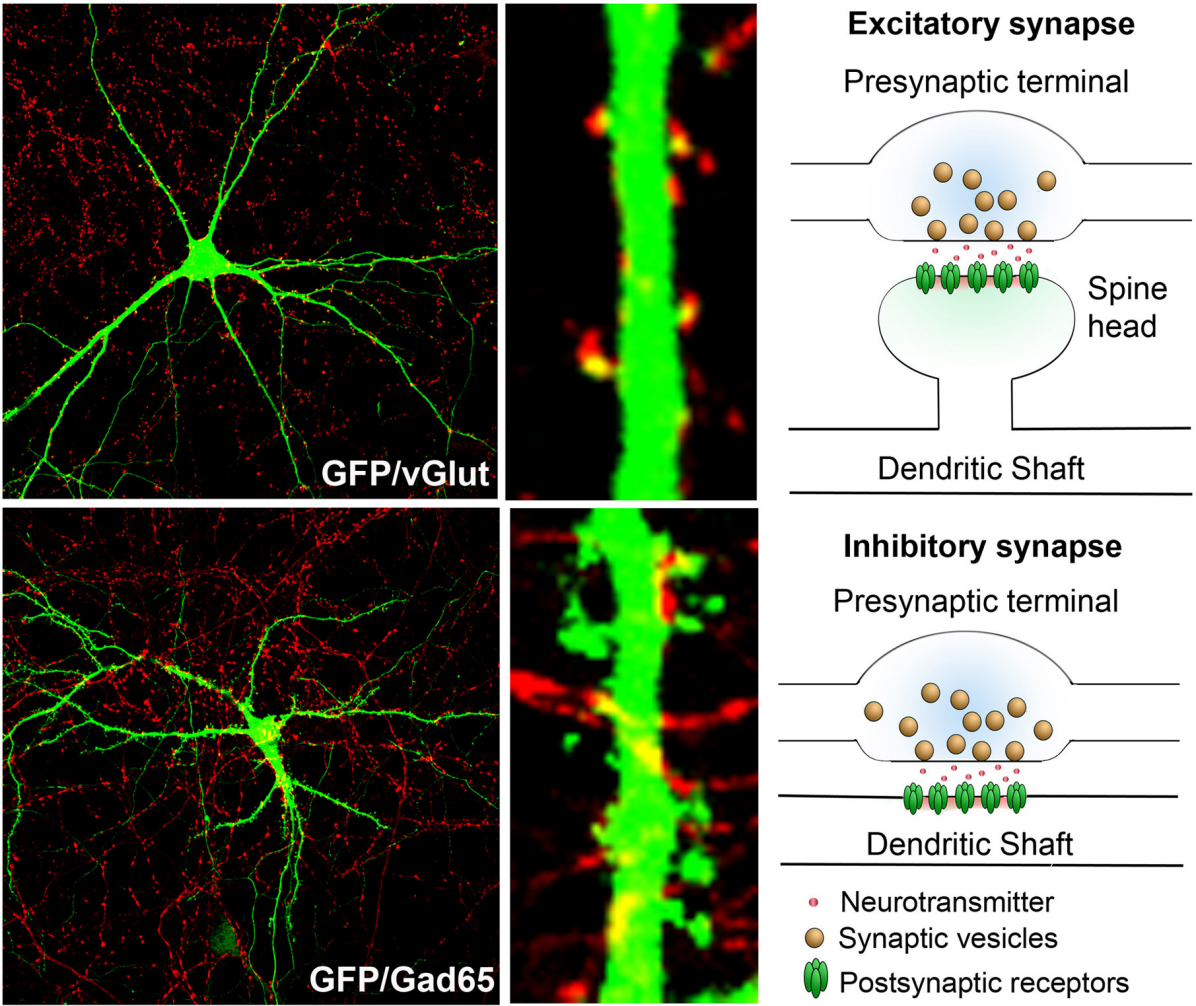
**Synaptic terminals on dendrites and schematic of excitatory and inhibitory synapses: rat hippocampal neurons in culture (DIV 17) transfected with GFP (green) and immunostained with markers that label presynaptic terminals of either excitatory (top, red—vGlut1) or inhibitory (bottom, red—GAD65) synapses.** Note that the excitatory synapses predominantly form on spine heads, while the inhibitory synapses are predominantly localized in the dendritic shaft. The presynaptic terminals contain neurotransmitter loaded vesicles, while the postsynaptic region harbors neurotransmitter receptors.

Axons harbor presynaptic terminals that contain neurotransmitter bearing vesicles. The presence of voltage gated sodium channels allows the propagation of action potentials along the length of the axon to the presynaptic terminals, thus promoting neurotransmitter release. Dendrites bear synapses, both excitatory and inhibitory (Figure 1). Synapses represent major sites of information input into neurons.

The structure and arborization of dendrites has a profound impact on the processing of neuronal information. Most of the pyramidal neurons in the cortex and hippocampus have a characteristic pyramidal morphology with distinct large apical and shorter basolateral dendrites that originate from the apex and base of the soma respectively, each with multiple branches. Cortical pyramidal neurons are organized into six layers and neurons from each layer have characteristic dendrite branching patterns, coincident with their functional roles (Spruston, 2008). Synapses are the major sites of information input into neurons. The pattern of dendritic arborization is critical because it determines the synaptic input field of the dendrite. Distinct dendritic regions receive synaptic input from different sources. For example, the CA1 neurons in the hippocampus receive their input at the apical tuft dendrites from the entorhinal cortex, while the basal dendrites receive their input from the CA3 neurons. In addition to functional synapses, dendrites also harbor a variety of receptors, channels (Lujan et al., 2009) and signaling molecules, including extrasynaptic NMDA (Petralia et al., 2010; Papouin et al., 2012) and GABA (Kasugai et al., 2010) receptors, that regulate various aspects of neuronal function (Ratnayaka et al., 2011), including cell death. Emerging evidence suggests that these non-synaptic receptors may also play key roles in the transmission of neuronal information (Ratnayaka et al., 2011). In contrast to the classical view, it is now clear that dendrites do not merely function in passive signal propagation but play key active roles in local signal computation (Larkum et al., 2009; Branco et al., 2010; Branco and Hausser, 2011; Emoto, 2012; Kim et al., 2012). The integration of presynaptic and postsynaptic events is strongly influenced by their location along the dendritic arbor. Postsynaptic elements at distal synapses have the highest gain for integration, while presynaptic elements located at the distal end have the least gain (de Jong et al., 2012), thus the length of the dendritic arbor is critical for signal integration. Dendritic functional roles are also critical in the storage of long-term memory (Govindarajan et al., 2011). Inhibitory interneurons form the other major type of neurons in the cortex and hippocampus (Mendez and Bacci, 2011) and are not as homogeneous as the pyramidal neurons. Inhibitory neurons can be further classified into various subclasses. Inhibitory interneurons also have extensive dendritic arbors which have integral roles in neuronal computation (Goldberg and Yuste, 2005; Camire et al., 2012), however most of them do not harbor spines or have relatively low spine densities. Thus mechanisms that regulate the initiation, formation and maintenance of dendritic arbors can have a profound influence on the formation and maintenance of neural circuits and consequently higher order brain functions, including learning and memory.

## Development of dendritic polarity

During development of the cortex and hippocampus, neurons are specified, exit the cell cycle and undergo migration that eventually allows them to reach their specified destination and form connections with other neurons (Marin et al., 2010). *In vivo*, most of these neurons undergo polarization during migration eventually leading to a highly polarized neuron with extensive axonal arbors and dendritic trees. Axons and dendrites are structurally and functionally distinct and these distinctions are maintained over the lifetime of the neuron. One of the central challenges in neurobiology has been to determine the molecular mechanisms that control the specifications of the axon vs. the dendrite. Over the last several years, it has become apparent that several extrinsic and intrinsic factors cooperate to determine the initiation of neuronal polarity. While several molecular effectors that control the specification of axons, including PI3-kinase, PTEN, GSK3β, the Par family of proteins and the SAD kinases (Barnes et al., 2007; Barnes and Polleux, 2009; Shelly et al., 2011) have been characterized, much less is known about how dendrite specification is achieved. Moreover, dendrites are not homogeneous structures and it is unclear what the extrinsic and intrinsic cues are that control dendritic polarity to allow the initiation, development and maintenance of apical and basolateral and other lateral dendrites. Given the roles of molecular effectors, local protein degradation, cytoskeletal dynamics, and molecular motors in the specification of axons (de Anda et al., 2005), it is likely that similar cues specialized for dendrites exist and remain to be identified and characterized. Interestingly, a subset of neuronal storage disorders with increased levels of GM2 ganglioside result in the reinitiation of ectopic dendrites that form connections on mature neurons in the cortex (Siegel et al., 2002), suggesting that molecular cues exist that control dendrite initiation separately from axon initiation and dysregulation of such mechanisms could have pathological consequences (Cox and Cachon-Gonzalez, 2012).

## Dendritic synapses

In the hippocampus and cortex, the majority of excitatory synapses are formed on spines while inhibitory synapses are predominantly formed on the cell bodies and dendritic shafts. Spines are small protrusions that arise along the dendritic shaft. Structurally, these have a long thin neck and a head that bears the excitatory synapse (Figure 1), but can have varied morphology (Bourne and Harris, 2008). Postsynaptic synapses bear the synaptic machinery that can respond to neurotransmitters and convey information. Spines and synapses are the major sites of information input into the neurons. More importantly, they are the major sites of dendritic plasticity (Matsuo et al., 2008; Harnett et al., 2012; Sanders et al., 2012) and considerable effort has been and is being directed toward defining the molecular components of these structures and understanding their contribution to plasticity (Lai et al., 2012) and higher order brain functions, including learning and memory (Vanleeuwen and Penzes, 2012). Moreover, aberrations in synaptic structure and function are observed in a variety of neurodevelopmental, neuropsychiatric, and neurodegenerative disorders (Penzes et al., 2011; Yu and Lu, 2012). We refer the readers to other excellent reviews on the roles of synapses in plasticity (Bosch and Hayashi, 2012; Murakoshi and Yasuda, 2012; Okabe, 2012) and disease (Pavlowsky et al., 2012; Verpelli and Sala, 2012).

## Molecular control of dendrite arborization

The establishment of the dendritic arbor involves dendrite extension, addition, elongation, retraction, and pruning (Jan and Jan, 2010; Emoto, 2012). These processes are influenced by synaptic activity. In the adult, the dendritic arbor is mostly stabilized and is only induced to undergo remodeling under pathological conditions (Wu et al., 1999; McAllister, 2000).

Molecular mechanisms, both extrinsic and intrinsic, exist that allow for exquisite temporal and spatial control of arborization to ensure that the extensive dendritic arbor is correctly generated and maintained during the lifetime of the organism. Moreover, the functional roles of these molecules are intertwined and regulated to allow for tight control over dendrite extension and arborization.

In this review, we discuss the various classes of molecules that control dendritic arborization in hippocampal and cortical neurons with a few more recently identified examples from each class. While some of these mechanisms and pathways have also been implicated in dendrite morphogenesis in other types of neurons, discussing other types of neurons is beyond the scope of this review and we refer the readers to other excellent reviews (Jan and Jan, 2010; de la Torre-Ubieta and Bonni, 2011) on these topics.

**Figure 2:**
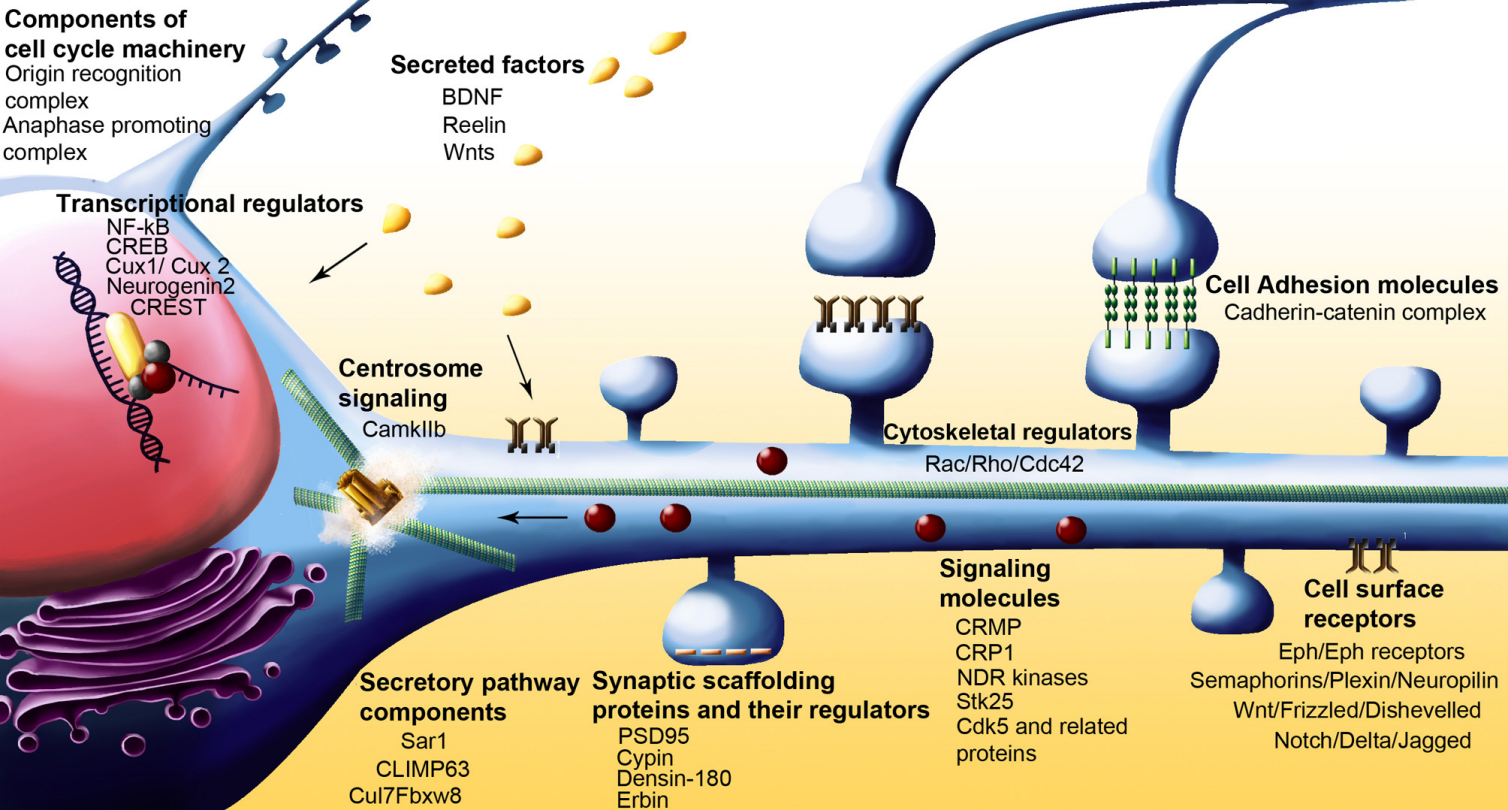
**Schematic of neuron showing various classes of molecules with some of the more recent examples that have been implicated in dendrite arborization**.

**Figure 3:**
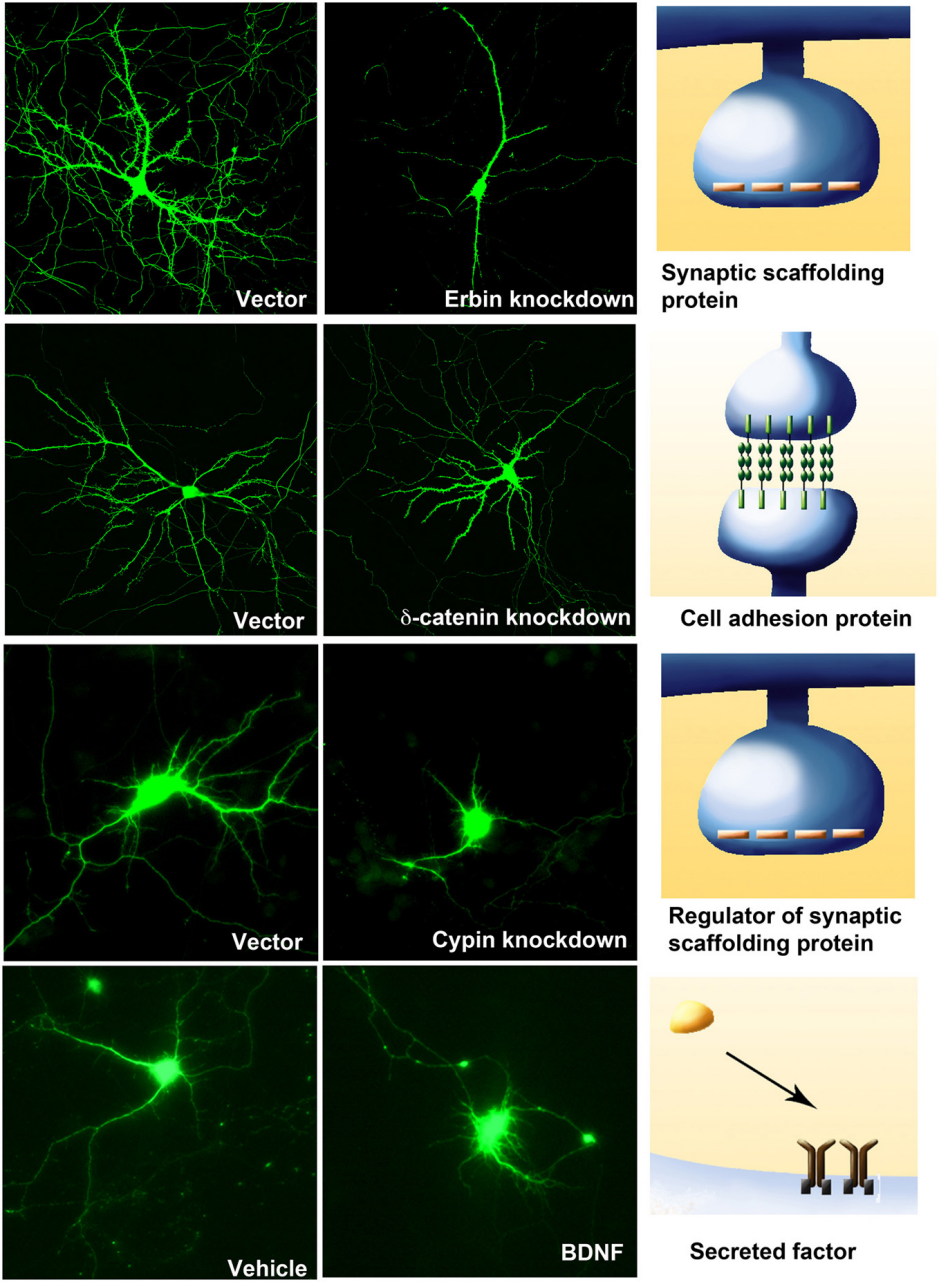
**Four examples of molecules from different classes that regulate dendritic branching.** Knockdown of the postsynaptic density proteins, Erbin (DIV 11–17) or regulator of postsynaptic proteins, Cypin (DIV 5–10), or a component of the Cadherin-Catenin cell adhesion complex, δ-catenin (DIV 11–17) result in a compromise in dendritic branching. However, the addition of BDNF (25 ng/ml at DIV 7–10) to hippocampal neurons increases neurite outgrowth and branching.

Several classes of molecules participate in ensuring correct dendritic architecture. These include secreted molecules, cell surface receptors, cell adhesion molecules, postsynaptic density proteins, signaling molecules, regulators of the actin cytoskeleton, molecules that control Golgi trafficking, calcium signaling proteins, and transcription factors. Table 1 while several of these proteins play important functional roles outside central neurons, it is becoming increasingly appreciated that they control multiple aspects of neuronal morphology, including dendritic branching (Figures 2 and 3).

**Table 1:** **Summary of molecules regulating dendritic morphogenesis**.

**Factor/molecule**	**Functional role**	**Role in dendrite morphogenesis**	**Components of pathway**	**References**
**SECRETED FACTORS, CELL SURFACE RECEPTORS, AND CELL ADHESION MOLECULES**				
BDNF	Neurotrophic factor	Increases dendrite branching	Cypin, MAPK, CREB	Yacoubian and Lo, 2000; Horch and Katz, 2002; Baj et al., 2011
Reelin	Extracellular matrix glycoprotein	Increases dendritic growth	VLDL receptor, apo E receptor 2, MAP1B, GSK313	Lambert de Rouvroit and Goffinet, 2001; Niu et al., 2004; Gonzalez-Billault et al., 2005; D'Arcangelo, 2006
Wnt family	Secreted glycoproteins	Promotes dendrite complexity	Frizzled, disheveled, Rac, Jnk, Par1b/MARK2	Rosso et al., 2005; Terabayashi et al., 2007
Ephrins and Eph receptors	Receptor tyrosine kinases and their ligands	Promotes dendrite arborization	KIF5, GRIP1	Klein, 2009
Semaphorins	Secreted and transmembrane proteins	Initial step of neuronal polarization and promotes the origin of dendrites, controls dendrite bifurcation and complexity of basal dendrites	Neuropilin, Plexin, CRMP, LKB1, GSK36, PI3 kinase, mTor	Nakamura et al., 2009; Tran et al., 2009; Vodrazka et al., 2009; Shelly et al., 2011
Notch	Cell surface receptor	Promotes dendritic complexity	Delta, jagged, Nf-κB	Breunig et al., 2007; Bonini et al., 2011
Cadherin-catenin cell adhesion complex	Cell adhesion complex, participates in signaling and regulation of actin cytoskeleton	N-cadherin, δ-catenin, p120ctn and β-catenin promote dendritic branching	Rac/Rho, erbin, Wnt, αN-catenin, Arp3	Elia et al., 2006; Arikkath et al., 2008; Tan et al., 2010
**SYNAPTIC SCAFFOLDING PROTEINS AND THEIR REGULATORS**				
Psd95	Postsynaptic density scaffolding protein	Stop signal for proximal dendrite branching	End-binding protein 3 (EB3)	Charych et al., 2006
Cypin	Protein that binds to the PDZ domains of PSD-95 and decreases localization of PSD-95 at the postsynaptic density	Promotes dendritic branching	Tubulin, RhoA, BDNF, Snapin	Firestein et al., 1999; Akum et al., 2004; Chen et al., 2005; Chen and Firestein, 2007; Kwon et al., 2011
LAP family of proteins	Densin-180 and Erbin— postsynaptic density proteins	Promotes dendritic branching	Shank, δ-catenin	Quitsch et al., 2005; Arikkath et al., 2008
**SIGNALING MOLECULES**				
Calmodulin kinase 2b	Calcium/calmodulin—dependent protein kinases	Inhibitor of dendrite extension	PCM1, cdc20	Puram et al., 2011
CRMP	Collapsin response mediator proteins	Suppression of inappropriate spatial dendrite bifurcation during the dendritic extension	Sema3A	Niisato et al., 2012
CRP1 (Cysteine rich protein 1)	Member of the cysteine-rich protein (CRP) family which is a subgroup of the LIM—domain protein family	Dendritic growth	Actin	Ma et al., 2011
NDR (nuclear Dbf2- related) kinases	Subclass of the protein kinase A/G/C family of kinases	Proximal dendrite growth and branching	AP2 associated kinase	Ultanir et al., 2012
Stk25 (Serine/Threonine Kinase 25)	Kinase	Length of apical and thickness of basal dendrite	?	Matsuki et al., 2010
CDK5 and related proteins	Kinases	Dendritic branching	Variety of soluble factors, cell surface receptors and other signaling molecules, including the Rho family of GTPases	Cheung and Ip, 2007; Cheung et al., 2007; Zhang et al., 2010; Fu et al., 2011; Su and Tsai, 2011
**REGULATORS OF THE CYTOSKELETON**				
Rac/Rho/Cdc42	Small GTP binding proteins	Activation of Rac and Cdc42 promote the extension of neurites, while activation of Rho mediates the retraction of neurites	P120, cypin, Arf6 and other signaling pathways	Hernandez-Deviez et al., 2002; Luo, 2002; Leemhuis et al., 2004; Elia et al., 2006; Chen and Firestein, 2007
**REGULATORS OF SECRETORY PATHWAY**				
Sar1	GTP binding protein that functions in trafficking from the endoplasmic reticulum to the golgi	Dendritic length	?	Ye et al., 2007
CLIMP63	ER integral membrane protein	Dendritic branching	Phosphorylation dependent microtubule association	Cui-Wang et al., 2012
Cul7^Fbxw8^	Cul7^Fbxw8^ forms an E3 ubiquitin ligase	Dendritic arbor and length	Grasp65, OBSL-1	Litterman et al., 2011
**COMPONENTS OF CELL CYCLE MACHINERY**				
Origin recognition complex	complex that allows for the initiation of DNA duplication during the cell cycle	Promotes dendritic growth and branching	?	Huang et al., 2005
Anaphase promoting complex (APC):	Ubiquitin ligase complex that controls cell cycle transitions via the coactivators Cdc20 and Cdh1	Dendrite initiation and maintainance	Cdc20, HDAC6, ld1	Kim et al., 2009
**TRANSCRIPTIONAL MECHANISMS**				
Nf-κB	Transcription factor	Promotes dendrite length and branching	Nerve growth factor, notch, jagged	Gutierrez et al., 2005; Bonini et al., 2011
CREB	Transcription factor that regulates gene expression through binding cAMP response elements	Promotes activity dependent dendritic growth branching	CamKIV, CamKK, MAP kinase pathway, Wnt2	Redmond et al., 2002; Wayman et al., 2006
Cux1	Homeobox transcription factor	Regulator of dendritic complexity	p27^Kip1^ RhoA	Li et al., 2010
Cux2	Homeobox transcription factor	Regulator of dendritic complexity	?	Cubelos et al., 2010
Neurogenin2	Basic-Helix-Loop-Helix (bHLH) transcription factor	Regulation of dendritic polarity	?	Hand et al., 2005
CREST (calcium- responsive transactivator)	Nuclear protein related to Syt.	Promotes calcium dependent dendritic growth	CBP (CREB binding protein), nBAF chromatin remodeling complexes	Aizawa et al., 2004; Wu et al., 2007

## Secreted factors, cell surface receptors, and cell adhesion molecules

Secreted factors can exert both paracrine and autocrine regulatory effects on dendritic outgrowth and extension. Receptors and cell adhesion molecules can participate in transducing external stimuli into intracellular signaling cascades and thus influence dendritic growth and branching.

### Brain-derived neurotrophic factor

Neurotrophins are neurotrophic factors that play key roles in dendritic growth and branching. Four members of the family include nerve growth factor (NGF), brain derived neurotrophic factor (BDNF) and neurotrophins 3 and 4 (NT-3 and NT-4). These factors exert their effects through binding the TrK family of receptors and the p75 neurotrophin receptor (p75NTR). Of these, BDNF is the most widely studied and the most critical factor regulating dendrite outgrowth and branching. Several studies have shown that BDNF application increases dendrite number in pyramidal neurons (McAllister et al., 1995; Horch and Katz, 2002), and these increases in dendrite branching act in a distance-dependent manner (Horch and Katz, 2002). Different isoforms of TrkB receptors show different effects on dendrite branching. Full length TrkB activation results in increased proximal dendrite numbers, whereas truncated T1 promotes net elongation of dendrites when overexpressed in cultured neurons (Yacoubian and Lo, 2000). BDNF binds to the TrkB isoforms and activates various signaling pathways, such as the Ras/MAPK cascade, PI3 kinase/Akt pathway, and IP_3_-dependent calcium release. Thus, they promote transcriptional changes for proteins that are involved in cell survival, death, cytoskeletal changes, and neurite outgrowth (Chao, 2003; Segal, 2003). Recent studies show that distinct 5'UTR BDNF transcripts differentially shape dendritic arbor in an area-specific manner by selective activation of TrkB receptors (Baj et al., 2011) suggesting that distinct mechanisms are involved in shaping dendrite complexity.

### Reelin

Reelin is a large, secreted extracellular matrix glycoprotein, secreted by Cajal-Retzius cells during early cortical development. Reelin is also expressed by interneurons through life (Liu et al., 2001). While the role of Reelin in the migration of cortical neurons has been well established, it is now clear that Reelin regulates multiple aspects of neuronal development and maintenance, including dendrite extension. Loss of Reelin leads to reduction in dendrite growth and this effect of Reelin is mediated by signaling through the very low density lipoprotein receptor and the apolipoprotein E receptor 2 (D'Arcangelo, 2006). Conversely, addition of reelin increases dendritic growth of hippocampal neurons (Niu et al., 2004) and induces MAP1B phosphorylation via activation of GSK3β (Lambert de Rouvroit and Goffinet, 2001; Gonzalez-Billault et al., 2005).

### Wnt family of proteins

The Wnt proteins are a family of secreted glycoproteins that bind the frizzled receptor and result in the activation of the scaffolding protein, Disheveled and subsequently the canonical or non-canonical pathways. In the canonical pathway, this activation is followed by activation of GSK3β, a serine-threonine kinase, which in turn activates β-catenin mediated transcription. In the non-canonical pathway, the Wnt proteins can directly activate the Rho GTPases to modulate the actin cytoskeleton (Salinas and Zou, 2008). Wnt7b promotes dendritic complexity (Rosso et al., 2005) via the PDZ domain of Disheveled in association with Rac and Jnk, but independent of the canonical signaling pathway and Rho. In addition, Wnt-3a promotes the recruitment of Par1b/MARK2, a polarity regulating kinase, to the membrane which then promotes the phosphorylation of MAP2 which is selectively localized to dendrites (Terabayashi et al., 2007). Interestingly, the expression of another Wnt, Wnt2, that also promotes dendrite arborization is regulated by synaptic activity (Wayman et al., 2006), suggesting that Wnts may play key roles in the regulating dendrite morphogeneis in an activity dependent manner.

### Ephrins and Eph receptors

The Eph receptor tyrosine kinases and their ligands, the ephrins, play critical roles in various aspects of axon and synapse development and function. The Eph receptor tyrosine kinases are subdivided into two major families, the EphA and EphB. The ephrin ligands are anchored to the plasma membrane via a GPI linked anchor (Ephrin-A) or transmembrane domain (Ephrin-B). Interestingly, the Eph-Ephrin signaling is bidirectional and both forward and reverse signaling mechanisms are important in various aspects of CNS development and function (Klein, 2009). Indeed, loss of EphB1, EphB2 and EphB3 in a mouse model results in a reduction in dendritic arborization (Hoogenraad et al., 2005) in mature neurons, suggesting that Ephs play important roles in regulating dendrite arborization. This can be partly attributed to the necessity of EphB to be trafficked to the surface membrane via a KIF5 (a microtubule motor protein) and GRIP1 (glutamate receptor interacting protein 1) dependent mechanism (Hoogenraad et al., 2005).

### Semaphorins

Semaphorins form a large family of a group of proteins that include both secreted and transmembrane proteins. The major receptors for Semaphorins are members of the Plexin family of transmembrane proteins. The Neuropilins may also serve as coreceptors for semaphorins. While several members of the family play critical roles in axon guidance (Kolk et al., 2009) and spine morphogenesis (Tran et al., 2009) and dendrite guidance, it is now becoming clear that multiple semaphorins also regulate dendrite outgrowth and architecture at different stages during development. Recent studies indicate that Sema3A regulates the initial step of neuronal polarization and promotes the origin of dendrites (Shelly et al., 2011), while suppressing axon formation, both *in vivo* and *in vitro*. Mechanistically, Sema3A promotes the elevation of cGMP, but reduces the levels of cAMP and activity of protein kinaseA. Interestingly, loss of Sema3A increases the bifurcation of dendrites within the stratum pyramidale (Nakamura et al., 2009). Sema3A also promotes the complexity of basal dendritic arbors via neuropilin2 and PlexinA3 (Tran et al., 2009). Sema4D promotes dendritic branching in hippocampal neurons via the PlexinB1 receptor, PI3 Kinase and mTor pathways (Vodrazka et al., 2009).

### Notch

Notch is a cell surface receptor that when cleaved releases an intracellular fragment that translocates to the nucleus and regulates gene expression. Two families of ligands, Delta and Jagged are known to activate Notch. Signaling via the Notch receptor has an inhibitory effect on dendrite outgrowth. In cortical neurons, overexpression of a dominant negative Notch or knockdown leads to decreased dendritic branching (Breunig et al., 2007; Bonini et al., 2011) Overexpression of Notch leads to decreased average dendritic length, but increased complexity. Interestingly ablating *in vivo* expression of Notch in glia using genetic techniques results in a compromise in the dendritic complexity of newborn neurons in maturing hippocampal neurons (Breunig et al., 2007) suggesting that a complex role for Notch in regulating dendritic branching *in vivo*.

### Cadherin-catenin cell adhesion complex

The Cadherin-Catenin cell adhesion complex, comprising the transmembrane domain containing protein Cadherin with its associated cytosolic Catenins, mediates cell adhesion. It is now clear Cadherins and Catenins play critical roles in regulating the dendritic arbor. N-Cadherin is required for activity dependent increase in dendrite arborization, a functional role that requires it to regulate the actin cytoskeleton via the cytosolic αN catenin and Arp3 (Tan et al., 2010). δ-catenin, and P120ctn, both cytosolic catenins that bind to the juxtamembrane domain of cadherin, also play critical roles in regulating dendrite arborization although through different regulatory pathways. Loss or knockdown of P120ctn leads to a decrease in dendritic branching. This is accompanied by an increase in active RhoA and decrease in active Rac suggesting that these small G proteins play critical roles in the ability of p120ctn to regulate dendrite branching (Elia et al., 2006). However, loss of δ-catenin reduces dendrite branching with no effects on the levels of active Rac and RhoA, suggesting that p120ctn and δ-catenin utlize different mechanisms to regulate dendrite branching. Consistently, Erbin, a LAP family protein is known to be an upstream regulator of δ-catenin in regulating dendrite branching and functions by promoting proper localization of δ-catenin (Arikkath et al., 2008). Additionally, β-catenin overexpression promotes activity dependent dendrite branching through a Wnt dependent pathway.

## Synaptic scaffolding proteins and their regulators

A number of synaptic scaffolding molecules, predominantly localized to postsynaptic densities, have been identified to be critical regulators of dendritic morphogenesis. A number of these appear to influence dendritic arborization by directly regulating the actin and microtubule cytoskeleton.

### PSD-95 (postsynaptic density protein-95)

PSD-95 is a crucial scaffolding protein, which assembles signaling complexes at excitatory synapses. Its protein-protein interaction motifs, called PDZ domains (Kim and Sheng, 2004), bind to other PDZ domains and to various receptors and ion channels that contain the C-terminal PDZ binding consensus sequence T/S-X-V/I. The regulation of PSD-95 expression/degradation, clustering, and localization is important for neurons establishing and maintaining proper synaptic connections (Matus, 2000). In addition to its synaptic function, PSD-95 also exhibits a nonsynaptic function during hippocampal neuron dendritic development (Charych et al., 2006). PSD-95 acts as a stop signal for proximal dendrite branching by disrupting microtubule organization (Charych et al., 2006; Sweet et al., 2011). PSD-95 directly interacts with a proline-rich region of end-binding protein 3 (EB3) by its src homology 3 (SH3) domain, and alters microtubule structure (Sweet et al., 2011). Taken together, PSD-95 regulates dendritic morphology in young neurons.

### Cypin (cytosolic PSD-95 interactor)

Cypin was originally identified as a protein that binds to the PDZ domains of PSD-95 and decreases localization of PSD-95 at the postsynaptic density (Firestein et al., 1999). It localizes to spine necks and dendrites. Cypin increases dendrite branching when overexpressed in hippocampal neurons. Cypin directly binds to tubulin heterodimers and promotes microtubule assembly (Akum et al., 2004), and thus regulates dendrite branching via cytoskeletal rearrangement. In addition, Snapin antagonizes tubulin binding to cypin, thus changing dendrite branching patterns (Chen et al., 2005). RhoA controls dendrite branching by regulating cypin protein levels (Chen and Firestein, 2007). Moreover, BDNF increases proximal dendrites by CREB-dependent transcriptional activation of cypin (Kwon et al., 2011).

### Members of the LAP family of proteins

Two members of the LAP (leucine rich and PDZ domain containing) family of proteins, namely erbin and densin-180 that localize to synapses have been implicated in dendritic branching. Knockdown of erbin reduces total dendritic length, while overexpression of densin-180 promotes dendritic branching and complexity (Arikkath et al., 2008). While erbin regulates dendritic branching through δ-catenin, a component of the cadherin-catenin cell adhesion complex, the effects of densin-180 are mediated by shank and possibly δ-catenin (Quitsch et al., 2005).

## Signaling molecules

Signaling molecules participate in dendritic branching, at least in part by regulating the cytoskeleton. They may also function as transducers of information from cell surface receptors to control dendrite arborization. While examples of individual signaling molecules that control dendrite arborization are known, it is not always clear what the regulators that control these signaling pathways are.

### Calmodulin kinase IIB

The calcium/calmodulin-dependent protein kinases (CaMKs) in the brain are predominantly composed of the α and β isoforms which function by forming homo or heterodimers. Knockdown of CamkIIβ leads to an increase in dendrite extension throughout development. This functional role is independent of CamKIIα and CamkIIβ controls dendritic branching by localizing at the centrosome via its association with PCM1, a centrosomal targeting protein. At the centrosome, CamkIIβ phosphorylates cdc20 (coactivator of APC), thus inhibiting its activity and leading to dendrite retraction (Puram et al., 2011).

### CRMP (collapsin response mediator proteins)

The CRMPs are a family of cytosolic phosphoproteins. The family consists of five known members namely CRMP 1–5. Some of these members have been implicated in the signaling mechanisms underlying Sema3 mediated axon guidance. Recent studies however suggest that their functional roles extend to the control of specific aspects of dendrite arborization. In mice that are genetically null for CRMP4, hippocampal pyramidal CA1 neurons have increased bifurcation in the stratum pyramidale layer, suggesting a critical role for CRMP4 in suppression of inappropriate spatial dendrite bifurcation during the dendritic extension (Niisato et al., 2012). In addition, loss of CRMP4 leads to an increase in dendritic branch points. These effects are mediated, at least in part, by Sema3A since loss of CRMP4 leads to a compromise in the ability of Sema3A to promote dendritic branching.

### Cysteine-rich protein1

CRP1 is a member of the cysteine-rich protein (CRP) family which is a subgroup of the LIM-domain protein family. Knockdown of CRP1 leads to a decrease in dendritic growth during development, while overexpression does not alter the total neurite length. This functional ability is mediated in part by its actin bundling activity (Ma et al., 2011).

### NDR (nuclear Dbf2-related) kinases

The NDR kinases are a subclass of the protein kinase A/G/C family of kinases. The NDR family of proteins control cellular morphognesis in various cell types, however recent evidence indicates that NDR1 and 2 contribute to dendrite morphogenesis. The activity of both NDR1 and 2 inhibits proximal dendrite growth and branching. These effects are mediated by the AP2 associated kinase, which functions downstream of NRD1/2 in regulating dendritic branching (Ultanir et al., 2012).

### Stk25 (serine/threonine kinase 25)

Knockdown of stk25 promotes an increase in the length of the apical dendrite coupled with increased thickness of the basal dendrite (Matsuki et al., 2010).

### Cdk5 and related proteins

Cdk5 is a serine/threonine kinase that functions in multiple aspects of neuronal development. The activation of cdk5 is dependent on two activators, p35, and p39, which are predominantly expressed in neurons (Cheung and Ip, 2007). Cdk5 regulates neurite outgrowth by cooperating with a variety of soluble factors (Cheung et al., 2007), cell surface receptors and other signaling molecules, including the Rho family of GTPases (Su and Tsai, 2011). Knockdown or inhibition of the kinase activity of cdk5 inhibits dendrite growth and branching while inhibition of its ability to be S-nitrosylated increases dendritic branching (Zhang et al., 2010). Pctaire1, a cyclin-dependent kinase (Cdk)-related protein, also plays a key role in regulating dendrite arborization, both during development and at later stages. This functional role is mediated by its ability to be phosphorylated by cdk5 and its ability to function as a kinase (Fu et al., 2011).

## Regulators of the cytoskeleton

Modulation of the cytoskeleton is key in eliciting structural changes in dendrites. Consequently, regulators of the cytoskeleton play a key role in dendrite arborization.

### Rac/Rho/Cdc42

Rac, Rho, and Cdc42 are included in a family of small GTPases.

Signaling by one or more of these small GTPases appears to play a role in dendrite initiation, growth and dendrite branching (Luo, 2002; Leemhuis et al., 2004). Activation of Rac and Cdc42 promote the extension of neurites, while activation of Rho mediates the retraction of neurites. These small GTPases also cooperate with other signaling pathways to regulate dendrite arborization, including a component of the cadherin-catenin cell adhesion complex (Elia et al., 2006), a postsynaptic density component, cypin (Chen and Firestein, 2007) and a member of the ADP-ribosylation factor (ARF) family of small GTPases, Arf6 (Hernandez-Deviez et al., 2002). These small GTPases function through a variety of complex molecular pathways (de Curtis, 2008; Chen et al., 2012) to control dendrites and other aspects of neuronal morphology and function and have been implicated in neurological disorders (Nadif Kasri and Van Aelst, 2008).

## Regulators of secretory pathway

In neurons, the secretory pathway that comprises the endoplasmic reticulum Golgi and post Golgi intermediates and ER and Golgi outposts contribute to the dendritic plasma membrane. Consequently proteins that control various aspects of secretory trafficking have been implicated in dendritic arborization (Ye et al., 2007).

### Sar1

Sar1 is a GTP binding protein that functions in trafficking from the endoplasmic reticulum to the Golgi. Knockdown of Sar1 during dendritic development (Ye et al., 2007) reduces the total dendritic, but not axon length, indicating that the secretory trafficking from the Golgi is an important contributor to the dendritic membrane.

### CLIMP63

CLIMP63 is an ER integral membrane protein. Its binding to microtubules is regulated by PKC-dependent phosphorylation. Phosphorylation of CLIMP63 prevents association with microtubules. In neurons, expression of phosphorylation deficient form of CLIMP63 results in fewer proximal dendritic branches, while expression of phosphomimetic form or RNAi mediated knockdown promotes dendritic branching (Cui-Wang et al., 2012). These effects are directly related to the ability of CLIMP3 to regulate the complexity of the ER.

### Cul7^Fbxw8^ ubiquitin ligase

Fbxw8 is an F-box protein that assembles with the scaffold protein, Cul7. The Cul7^Fbxw8^ forms an E3 ubiquitin ligase that localizes to the Golgi apparatus in neurons. Recent studies indicate that it is required for growth and elaboration of dendrites in hippocampal neurons. Knockdown of *Fbxw8* or *Cul7* leads to a simplification of the dendritic arbor accompanied by a reduction in the total dendritic length and increased dispersion of the Golgi apparatus. The Obscurin-like 1, OBSL1, protein controls the localization of Cul7 to the Golgi apparatus. Further, Grasp65, a Golgi protein that regulates secretory trafficking, is a substrate for Cul7^Fbxw8^ in organizing the Golgi structure and dendrite elaboration (Litterman et al., 2011).

## Components of the cell cycle machinery

Hippocampal and cortical neurons are postmitotic, but surprisingly proteins that control various aspects of the cell cycle machinery have been detected in these neurons. Some of these have been implicated in dendritic branching, suggesting that evolutionary adaptations might promote the use of the same protein in different cellular contexts thus promoting optimal utilization of existing resources.

### Origin recognition complex

The origin recognition complex is composed of a hexameric protein complex that allows for the initiation of DNA duplication during the cell cycle. However, it has recently become evident that components this complex are expressed in postmitotic adult hippocampal and cortical neurons. Knockdown of the Orc3 in hippocampal neurons leads to a severe reduction in dendritic growth and branching, a functional role that is mediated by its Walker ATPase motif (Huang et al., 2005).

### Anaphase promoting complex (APC)

The APC is an ubiquitin ligase complex that controls cell cycle transitions via the coactivators Cdc20 and Cdh1. It is now evident that the complex controls dendrite arborization in developing and mature neurons. Knockdown of cdc20 at early or late stages of development leads to a simplification of the dendritic arbor accompanied by a decrease in total dendritic length, suggesting that cdc20 controls both dendrite initiation and maintenance. Interestingly, the localization of cdc20 at the centrosome is critical for its ability to regulate dendrite development. Its functional role is promoted by an interaction with HDAC6, a class IIb histone deacetylase, while the helix-loop-helix protein Id1 is a substrate for the complex (Kim et al., 2009). The APC/cdc20/HDAC6/Id1 is one of the few known pathways that operate at the centrosome to control dendrite extension.

## Transcriptional mechanisms

Transcriptional mechanisms are key in regulating dendritic outgrowth, since such mechanisms can promote long-term changes by controlling gene expression programs comprising of several genes. While several transcription factors that regulate dendrite arborization have been identified, the challenge has been to identify the cues that activate these factors and delineate the gene expression programs that they coordinate.

### Nf-κB

Nuclear factor kB (NF-κB) is a ubiquitously expressed transcription factor, whose functional roles vary with cell context and dimerization (Gutierrez and Davies, 2011). The family consists of five related proteins, c-Rel, RelB, p65, p50, and p52 that function by hetero or homo dimerization and regulate gene expression positively or negatively by binding to elements in the promoter or enhancer regions of target genes. The iκB family of proteins functions to bind the NF-κb and hold them in an inactive cytosolic location. Activation of NF-κB is achieved by the removal of the bound iκB and subsequent translocation of the activated NF-κb to the nucleus. In neurons, the most common form of NF-κb is the p65/p50 heterodimer. Inhibition of NF-κB signaling or transcriptional activation reduces dendrite length in cortical pyramidal layer2/3 neurons (Gutierrez et al., 2005). Similarly, dendrite branching is compromised in cortical neurons from P50 null mice (Bonini et al., 2011). There appear to be at least two signals that use NF-κB as a mediator to regulate dendritic arbors. Addition of NGF to hippocampal neurons in culture reduces the number of primary dendrites and promotes the elongation of dendrites. Inhibition of NF-κB signaling perturbs these effects of NGF, suggesting that NF-κB is a downstream regulator of NGF induced dendrite arborization. In addition, Nf-κB also functions in the Notch signaling pathway to regulate dendrite arborization. Loss of the p50 subunit of NF-κB is accompanied by an increase in the expression of Notch and Jagged1. Inhibition of Notch restores the dendritic arborization deficits in the P50 null neurons, suggesting that Notch/Jagged1 function through NF-κB to regulate dendrite branching (Bonini et al., 2011). However, the transcriptional targets of NF-κB that control dendrite arborization are not well defined.

### CREB (cyclic AMP response element-binding)

CREB is a transcription factor that controls gene expression via binding to cAMP response ele*ments* (CRE). CREB is critical for activity induced dendritic growth and branching. This functional role is mediated in part by the ability of CREB to serve as a downstream effector of CamKIV (Redmond et al., 2002) and CaM-dependent protein kinase kinase via components of the MAP kinase pathway and regulate the expression of Wnt2 (Wayman et al., 2006).

### Cux1

The Cux proteins are a family of homeobox transcription factors that control cell proliferation and differentiation. Of these, Cux1 and Cux2 are expressed in central neurons. Overexpression of Cux1, but not Cux2, simplifies the dendritic morphology while knockdown enhances dendritic complexity in cultured cortical neurons. In this cell culture model, Cux1 functions as a transcriptional repressor of p27^Kip1^, thus knockdown of Cux1 leads to increased levels of p27^Kip1^ which in turn regulates the small GTP binding protein, RhoA (Li et al., 2010). Interestingly, however, murine models lacking either Cux1 or Cux2 layers 2–3 cortical neurons have simplified dendritic morphology at P60 (Cubelos et al., 2010). *In utero* introduction of shRNAs to Cux1 and Cux2 also results in a decrease in dendritic complexity. It is unclear how these differences in results can be reconciled and may perhaps reflect the influence of other unknown factors that contribute to the functional roles of Cux1 and Cux2 in dendrite morphogenesis.

### Neurogenin 2

Neurogenenin 2 is a member of the basic-Helix-Loop-Helix (bHLH) transcription factors. The expression of mutant forms of neurogenin2 that either cannot be phosphorylated or cannot bind DNA in early migrating neurons results in a multipolar dendritic morphology, with no distinct apical dendrite (Hand et al., 2005).

### Crest (calcium-responsive transactivator)

CREST is a nuclear protein related to Syt. Loss of CREST in a murine model results in a severe compromise in calcium dependent dendritic growth (Aizawa et al., 2004). These effects are mediated by its ability to bind CBP (CREB binding protein) and the nBAF neuron-specific chromatin remodeling complexes (Wu et al., 2007).

## Dendritic architecture and neurological disorders

Consistent with the necessity of appropriate dendritic architecture for higher order brain functions, including learning and memory, deficiencies in the architecture of dendrites have been observed in a variety of neurodevelopmental, neurodegenerative and neuropsychiatric disorders (Kulkarni and Firestein, 2012). For example, decreased dendritic branching in CA1 and CA4 hippocampal neurons occurs in patients with autism and neurons from patients with Rett syndrome. Similarly, altered dendritic arborization and decreased expression of glutamate receptors have been observed in CA3 hippocampal neurons of patients with schizophrenia (Kolomeets et al., 2007). Specific dendritic alterations are seen in cortical neurons in those with Rett syndrome. Decreased dendrite number have also been observed in patients with Alzheimer's Disease (Couch et al., 2010), and Down Syndrome (Elston, 2003).

In addition to the neurodevelopmental and neurodegenerative disorders, other neurological disorders may be associated with alterations in the dendritic arbor that compromise higher order functions, including cognition. For example, recurrent seizures during development in mice lead to a suppression of the growth of dendrites in the CA1 region (Casanova et al., 2012) and may thus contribute to the cognitive deficits observed in childhood epilepsy. Pathological conditions associated with other conditions that lead to alterations in higher order brain functions, also cause alterations in dendritic architecture (Liston et al., 2006). For example, stress paradigms in rats results in the retraction of the apical dendrite and branch number (Radley et al., 2005). Administration of nicotine results in an increased dendritic complexity in the insular cortex, associated with enhanced dendritic length and addition of new dendritic branches (Ehlinger et al., 2012). Prenatal exposure to cocaine or heroin in mice results in an increase or decrease respectively in dendritic arbors in the somatosensory cortex and cause impairments in short-term spatial memory (Lu et al., 2012). Neuropathic pain, associated with depression and cognitive decline, in a rat model is associated with an increase in the length and number of branches of basal dendrites (Metz et al., 2009). Thus, an abnormality in the dendritic arbor is a common theme seen in disorders of the central nervous system and these aberrations may be directly linked to deficiencies in higher order brain functions.

## Summary, outstanding questions, and challenges

A variety of different factors contribute to create and maintain the complex dendritic architecture that is key to higher order brain functions, including learning and memory. Each of these factors do not function in isolation, but cooperate with one or more of the other known or unknown molecules in a complex manner to control the formation and maintenance of dendritic architecture. For example, δ-catenin, a component of the cadherin-catenin cell adhesion complex regulates dendritic architecture during development via its functional interaction with an upstream activator, erbin (Arikkath et al., 2008). It is highly unlikely that erbin is its only upstream activator. In addition, it likely interacts with a variety of other unknown cellular factors to control the actin cytoskeleton to ultimately affect dendritic arbors. These factors remain unknown. It is also likely that various classes of these proteins interact with each other dynamically, so that their interactions and functional roles are aligned with the developmental requirements for sculpting or adult requirements for maintaining the dendritic arbor. In addition, these interactions and functional roles are likely regulated in an activity dependent manner to allow for dynamic control of dendritic architecture aligned with neuronal activity. Localized interactions of some of these classes of proteins may also influence specific architectural alterations, for example the generation and maintenance of secondary dendrites.

Thus the generation, sculpting and maintenance of the dendritic arbor is a complex process that requires coordination and cooperation of various classes of proteins. Many of these proteins and their mechanisms of action and their complex interactions with other known and unknown factors probably remain undefined. Future efforts would likely be directed at examining and defining these complex interactions and their roles in detecting and transducing information to the cytoskeleton to contribute to the generation and maintenance of the dendritic arbor.

It is now clear that the structure of the dendritic arbor is critical for signal integration, appropriate synapse formation and neural circuit function. It is also clear that several different classes of proteins play critical roles in determining the initiation and maintenance of the dendritic arbor. Many more probably remain to be identified. Moreover, dendritic arbors are not homogeneous structurally and functionally and it is evident that several mechanisms operate to control various aspects of branching. For example, molecular effectors that regulate either more proximal or more distal branching are known. Mechanisms that control the specialization of the apical vs. basolateral dendrites exist. Many challenges, however, remain in our understanding of the molecular control of dendritic arborization. What are the signals that allow initiation of dendrite formation? How are these integrated with neuronal migration? What are the key signals that coordinate the formation of the apical vs. basal dendrite? How are these dendritic polarities maintained through the lifetime of the animal? What are the factors that control layer specific branching of the dendritic arbor and prevent inappropriate branching? How do the different molecular pathways cross-talk to ensure the generation and maintenance of appropriate arborization? How are all these events coordinated with neuronal activity and neural development? Can manipulation of these pathways provide therapeutic avenues for neurodevelopmental disorders and acute neurological disorders that result in neuronal loss or compromise in neuronal architecture and function?

We are now in an exciting technological age where powerful tools are becoming increasingly available to address fundamental problems in neurobiology. These include tools that allow for genetic manipulation of murine models, control of neuron specific activation or inhibition *in vivo*, for example using optogenetic tools (Tye and Deisseroth, 2012), advanced imaging molecules (Weimer et al., 2012) and techniques and high efficiency gene sequencing techniques. These tools should allow us to develop new and critical insights into how the dendritic arbor develops and is maintained through life, how dendritic arborization contributes to neural circuit formation and function and how aberrations in dendritic arbors contribute to the pathology of various neurological disorders.

### Conflict of interest statement

The author declares that the research was conducted in the absence of any commercial or financial relationships that could be construed as a potential conflict of interest.
